# Afrocentric frameworks in recreation and leisure research: a perspective article

**DOI:** 10.3389/fspor.2024.1491824

**Published:** 2024-11-29

**Authors:** Michael Sakala

**Affiliations:** Physical Activity, Sport and Recreation (PhASRec), Health Sciences, North-West University, Potchefstroom, South Africa

**Keywords:** afrocentric, recreation, leisure, theoretical framework, ubuntu

## Abstract

This perspective article re-imagines and proposes key Afrocentric frameworks that can contribute towards animating the recreation and leisure discourse in Africa. The article foregrounds Afrocentricity, Sankofa, African Social Ontogenesis and Ubuntu as pertinent lenses through which recreation and leisure phenomena can be explored to respond to African realities. The article analytically draws on relevant scholarship to describe and interpretively glean implications for recreation and leisure research. The article's contribution to the discourse lies in its call for a re-think on how recreation and leisure research should engage with African realities.

## 1 Introduction

Scholarly research in the Global South is entreated to deploy contextually-responsive frameworks in exploring phenomena (1). African recreation and leisure research should amplify context-specific perspectives, instead of rehashing universalistic views if it is to be effective (2, 3). In this perspective article, I foreground Afrocentricity, Sankofa, Social Ontogenesis and Ubuntu as frameworks that can enrich recreation and leisure discourses in African contexts. Recreation and leisure are relatively young disciplines, with approximately 10 countries in Africa offering them as distinct disciplines in universities (4, 5). Mostly, recreation and leisure as fields of study are subsumed or fragmented under fields such as, *inter alia*, education, event management, geography, psychology, sociology, sport, hospitality and tourism (6, 7).

Notable recreation and leisure challenges in most African contexts include inadequate leisure education frameworks and ineffective policies (8). Africa is not a monolithic entity. It consists of 54 countries with numerous cultural and linguistic diversities (8). Kwame Nkrumah, a foremost pan-Africanist, argued that what unites Africans is greater than the differences. Ali Mazrui, an African Studies intellectual posited that the shared histories and experiences of colonisation are inescapable markers of a shared African identity (9). It is therefore beyond the scope of this perspective to prescriptively delve into the minutiae of how individual African cultures or regions can apply these frameworks. The perspective article serves as a general inducement to trigger conversations for recreation and leisure scholarship to optimally adopt Afrocentric perspectives in research practices.

## 2 Synopsis of afrocentric frameworks for recreation and leisure research

This section draws axiological, epistemological, ontological and methodological implications that may inform recreation and leisure research in African contexts. Axiology refers to value systems which guide research (10). Epistemology are explanations on how knowledge is conceived and produced to understand reality (11). Ontology refers to beliefs and assumptions on the nature of reality, while methodology refers to procedures undertaken in conducting research (10).

### 2.1 Afrocentricity

Afrocentricity is the analysis of phenomena from standpoints that centre the agency of Africans in societal transformation (12). Afrocentricity was developed by Molefi Asante to reposition African discourses by centring Africans and their experiences in research practices (13). It arose out of the inadequacy of Eurocentric models to enunciate the social realities of African societies (14). Its key principles are that Africans must look at knowledge from an African perspective and be central in setting discourse narratives (15).

#### 2.1.1 Axiological implications

The affirmation of African dignity is an axiological keystone of Afrocentricity (15). Western philosophers such as Georg Wilhelm, Friedrich Hegel and David Hume argued that Africans were sub-human. However, Afrocentricity's axiological effect is valorizing Africanness and its cultures (16). This translates to fairness and cultural sensitivity in integrating and interpreting African cultural beliefs, experiences and norms in research initiatives (17). For recreation and leisure research, this centres the place of Africans' agency in the discourse.

#### 2.1.2 Epistemological implications

The epistemological implications of Afrocentricity involve affirming contextualised constructions of knowledge by Africans (18). African perspectives as forms of knowledge should not only be centred, but must be understood from the viewpoint of the researched (19). For recreation and leisure research, this generates nuanced forms of knowledge which evolve authentic African recreation and leisure interpretations and illustrations.

#### 2.1.3 Ontological implications

Afrocentricity views reality as a multiplicity of worlds, which evokes a multifaceted way of regarding phenomena (20). The implications are that Africans should produce change through their own interpretation and construction of reality (21). For recreation and leisure, research should seek deeper understandings of socio-cultural dynamics to develop culturally responsive programmes.

#### 2.1.4 Methodological implications

Afrocentricity is associated with cultural and social immersion as opposed to scientific distance (22). It dovetails with research methods such as participatory action research, considered to be liberating and empowering for social change and envisioning alternative systems for Africans (23). In recreation and leisure, this may induce community involvement in all stages of research processes for co-production of knowledge.

#### 2.1.5 Case example

Makaudze and Mkhatshwa (24) deployed Afrocentricity in a study of Swati riddles as forms of leisure. The adopted axiological posture involved incorporating a Swati person to be a co-author, which provided cultural agency and contextually accurate interpretation of phenomena. The epistemic significance was that it allowed for knowledge to be generated from the viewpoint of one who understood the culture. Methodologically, the qualitative nature of the study provided in-depth and nuanced understandings and interpretations of riddles. However, since the study relied on secondary data, it had contextual limitations and possibilities of bias, thereby necessitating participatory methods involving people as primary sources of data to add multiple layers of nuance and voices.

### 2.2 Sankofa

Sankofa is an expression from the Twi speaking Adinkra tribe of the Akan people in Ghana (25). When divided into three syllables, namely “san” (return), “ko” (go), and “fa” (take), its English translation means return and fetch (26). The expression which captures the concept is “so wo were fi na wosan kofa a yenki”, which means, it is not taboo to return and get what you forgot (27). The Sankofa paradigm serves as a roadmap for the future by using the wisdom of the past in the present. Thus, African research practices cannot discount the past in its contribution towards knowledge based on heritage and tradition (25).

#### 2.2.1 Axiological implications

Sankofa is associated cultural humility, in which researchers respect and consider the histories of people and phenomena (28). Under Sankofa, research re-discovers lost identities and value systems, and legitimises indigenous knowledge as a body of scholarly thought (29). In the context of recreation and leisure research, aspects such as cultural tolerance, celebration of diversity and promotion of inclusivity should inform research practices (30).

#### 2.2.2 Epistemological implications

Sankofa dovetails with inductive knowledge generation which involves contextual understanding of phenomena and of the past (20). Traditional African communities, especially the elderly, are deemed key indigenous knowledge holders and transmitters (31). In recreation and leisure research, knowledge production may involve inter-generational transfer from the older generation to the younger, and re-imagining how such knowledge may be reclaimed to address current realities (32).

#### 2.2.3 Ontological implications

Under Sankofa, the view of reality implies a temporal connectedness of the past, present and future, as well as the integration of historical, the spiritual and cultural aspects (29). This results in nuanced understandings of reality, especially within Africa's diverse heritage (33). For recreation and leisure, discourses should explore and interpret phenomena from physical, social, spiritual, cultural and historical lenses.

#### 2.2.4 Methodological implications

Sankofa is associated with methodological flexibility wherein story-telling, proverbs and songs can be embedded in research activities as part of participatory research methods to amplify the voices of the researched (34). In addition to participatory methods, Sankofa opens up the space for recreation and leisure disciplines to delve into historical and archival research to unearth knowledge about past recreation and leisure practices. This further opens it up to interdisciplinary methods to provide holistic understandings of complex phenomena.

#### 2.2.5 Case example

A study by Madima (35) on cultural heritage preservation among the Venda people of South Africa deployed Sankofa. Its main axiological underpinning was social justice, involving reclaiming indigenous games knowledge. Epistemologically, the study generated knowledge inductively by recognising elderly community members to be custodians of indigenous knowledge. Ontologically, the study adopted a holistic view of reality by contextualising indigenous games within the broader milieu of Venda culture. In terms of methodology, the study adopted participatory action research which regarded participants as co-researchers. The study combined Sankofa with the Diffusions of Innovations theory, in which the former provided the basis for retrieving knowledge, while the latter explained how knowledge could be digitised. Such a combination provided a balance between tradition and modern innovation. However, it was not clear in the study on how Sankofa underpinned all steps of the research process, especially in data analysis.

### 2.3 African social ontogenesis

African Social Ontogenesis is a theory that explains African children's processes of development and identity through socio-cultural markers, and not merely biological indicators (36). It was developed by the Cameroonian Bame Nsamenang, who posited that society played an important role in childhood development by orienting African children to socially constructed developmental indicators (37). Key social milestones include child-naming ceremonies, social priming (teasing out socially appropriate responses from the child), as well as social apprenticing wherein children rehearse social roles (38). Through social ontogenesis, African children evolve social scripts in activities such as play to shape their own development with the help of significant others (39).

#### 2.3.1 Axiological implications

The central ethic of social ontogenesis is the recognition of communal relationships. The key implication is that research should map onto people's needs, social configurations and norms (29). By interpreting childhood development through socio-cultural lenses underpinned by biological indicators, the implication for recreation and leisure research is to ensure that a respectful appreciation of social relationships underpins research which intersects with childhood development.

#### 2.3.2 Epistemological implications

African Social-Ontogenesis inclines towards knowledge construction based on human interactions with socio-cultural context (19). This integrates diverse strands of knowledge threads into a unified conceptual system (as opposed to being in separate disciplines) (40), thus providing holistic insight into African-centred norms of competence (41). Recreation and leisure research, while maintaining its identity, may adopt multidisciplinary methods that break boundaries and foster deeper understandings of phenomena that involves complex social issues (6).

#### 2.3.3 Ontological implications

Under African Social Ontogenesis, reality is context-specific and is a construct of multiple influences (42). By not discounting biological factors in foregrounding socio-cultural factors, the theory adopts both an objective and subject view of reality (realism), in which objective biological facts are contextualised within subjective socio-cultural experiences (36). Recreation and leisure research may anchor on both objective and subjective notions of reality, with particular focus on developing context-specific understandings informed by social norms.

#### 2.3.4 Methodological implications

The social embeddedness of Social Ontogenesis predisposes it to research designs such as ethnography and phenomenology which provide for in-depth engagement with communities (43). Mixed methods approaches are also suitable to capture both objective and subjective realities (44). For recreation and leisure research, in addition to other proposed methods, adopting longitudinal methods may ensure prolonged community engagement and track social developmental milestones over time.

#### 2.3.5 Case example

Ejuu (45) deployed Social Ontogenesis principles to explore how indigenous games constituted social developmental activities in children's phases of growth in Uganda. In terms of axiology, study consent was obtained from the community at a clan meeting, where also parents and children consented to participate, thus pointing to the importance of communal and relational ethics. The study inclined towards constructivist methodology in which the social implications of indigenous games were examined. Methodologically, the study used observations, document analysis and interviews, which points to a realism ontological perspective that acknowledges the multifaceted nature of reality. However, as highlighted, longitudinal studies could provide more nuanced insight into how indigenous games constitute social markers over time.

### 2.4 Ubuntu

Ubuntu is a communitarian philosophy based on the African ideal of personhood, in which cooperating, empathy and collective judgements are key elements of the social order (20). Its other core tenets include caring, interdependence, sharing, solidarity, teamwork and unity (46). It is summarised in the Nguni expression “umuntu ngumuntu ngabantu”, whose transliteration is “a person is a person through other persons” (47). It reflects also in child rearing, wherein children are perceived to belong to the broader community, as aptly captured in the saying that goes “it takes a village to raise a child” (48). Nxumalo and Mncube (49) argue that play activities undergirded by Ubuntu philosophy stimulate children's critical thinking, creativity, collective values, and a sharing ethic.

#### 2.4.1 Axiological implications

Relational and reciprocity ethics undergird Ubuntu (29). The interests of the researched-upon are taken into account (46), hence the conduct of researchers must be aligned towards commitment to community, not only to self (50). In recreation and leisure, research experiences should be mutually beneficial and any spin-offs should also redound to the community and not only to the researchers.

#### 2.4.2 Epistemological implications

Under Ubuntu, knowledge is constructed and generated collaboratively (51). Ubuntu associates with inductive approaches in which dialogue, consultative processes and consensus-building form part of the knowledge generation process (28). In recreation and leisure research, Ubuntu may foster establishment of harmonious research teams in which co-creation and co-ownership of knowledge is fostered within a collaborative and mutually respectful process.

#### 2.4.3 Ontological implications

Ubuntu slants towards multifaceted and nuanced understandings of reality (51). The communitarian nature of life in most African societies necessitates that each member should be symbiotically embedded in the collective interests of the whole (52). For recreation and leisure, this means adopting a view of reality which incorporates multiple positions and insights within a communal discourse (6).

#### 2.4.4 Methodological implications

Ubuntu associates mostly with participatory and community research approaches (53). Collective sharing of ideas and decision making can be central to Ubuntu-underpinned research studies (46). Recreation and leisure research may consider adopting Ubuntu-underpinned methodologies which include linguistic inclusivity and co-opting research participants as co-researchers to build mutual trust, promote sharing of skills and gain insider cultural insight (54).

## 3 Summative critique

It is quite a telling indictment that my extensive search of literature could not yield an African-based recreation and leisure-related study that was clearly undergirded by Ubuntu. Research on recreation and leisure in African contexts spans across notable topics such as constraints to recreation and leisure participation, facility management, recreation therapy, adventure, benefits of participation, types or recreation and leisure activities, leadership practices and policies associated with recreation and leisure. A cursory view of most of the studies shows that they hardly deploy frameworks that can be regarded as Afrocentric to critically engage with African realities. While part of the reason could be attitudinal or even a lack of awareness, another reason could be the one by presented by Joseph et al. (55). In their study which explored a form of pretend play called *masekitlana* in South Africa, they consciously refrained from deploying any Afrocentric framework. By electing to stick to Vygotsky's theoretical ideas as opposed to, let's say Nsamenang, they reckoned that African frameworks tended to homogenise phenomena by veering towards nativism and cultural lock-in. While it is not the intention of this perspective to critique such a reductionist view, their standpoint provides some form of insight towards demystifying Afrocentric frameworks and avoiding excesses.

The common characteristic of the proposed frameworks is that their application is context specific. For example, while Ubuntu is attributable to Black Africans in most the regions of the Continent, different communities may emphasise certain aspects of values than others within the Ubuntu concept (56). The replicability of approaches may not be possible for comparative analyses as the frameworks may not subscribe to uniform templates. This also applies to the African Social Ontogenesis whose empirical grounding of the theory is based on impressionistic data from the Nso people of Cameroon. The social ontogenesis of children from a tribe in Cameroon cannot, in a wholesale manner, apply to the rest of the Continent. The same can be said about Sankofa whose ideas are extrapolated from a particular cultural group in Ghana.

Even though a number of examples provided herein involved indigenous activities, Afrocentric frameworks are not only applicable to themes associated with traditional or tribal practices. They span across various themes associated with modern technology and practices in recreation and leisure. This perspective is not an ideological or nostalgic call for the frameworks to be forcefully deployed even in circumstances or topics that may not apply. Afrocentric frameworks are not the only valid epistemic tradition for exploring phenomena going forward in the African context (16). It is a call for criticality which involves reflectively sifting through and refining the cultural past and presenting Afrocentric knowledge systems as viable alternatives in recreation and leisure disciplines (50).

## 4 Conclusion

In conclusion, this perspective article has argued that Afrocentricity, Sankofa, Social Ontogenesis and Ubuntu frameworks can augment recreation and leisure research in African contexts. Based on the deduced implications and the case analyses presented to illustrate this point, frameworks that are rooted in African thought and heritage provide axiological, epistemological, ontological and methodological insights that pertinently respond to African realities (57). The key implications drawn from the discussions underscore the importance of adopting Afrocentric knowledge systems as viable alternatives in recreation and leisure disciplines. Moving forward, this discussion presents a call for recreation and leisure scholarship in Africa to re-imagine nuanced, context-specific and culturally responsive research approaches towards informing theory, policy and practice (6). This will present a persuasive case for the continued relevance and growth of recreation and leisure disciplines in African contexts.

## Data Availability

The original contributions presented in the study are included in the article/Supplementary Material, further inquiries can be directed to the corresponding author.
